# Navigating silence and voice: South Asian women healthcare professionals in the UK NHS during COVID-19 and beyond – a qualitative study

**DOI:** 10.1136/bmjopen-2025-110607

**Published:** 2026-03-12

**Authors:** Saleema Kauser, Ana Paula Figueiredo, Bhavna K Pandya

**Affiliations:** 1 People Management and Organisations, The University of Manchester Alliance Manchester Business School, Manchester, UK; 2 The University of Manchester Alliance Manchester Business School, Manchester, UK; 3 Liverpool University Hospitals NHS Foundation Trust, Liverpool, UK

**Keywords:** COVID-19, QUALITATIVE RESEARCH, Health Services, HEALTH SERVICES ADMINISTRATION & MANAGEMENT, Awareness

## Abstract

**ABSTRACT:**

**Objective:**

To explore the experiences of South Asian female healthcare professionals in the UK National Health Service (NHS) during COVID-19, examining how the pandemic conditions exposed the ways in which race, gender and professional identity intersect to shape risk, silence and discrimination.

**Study design:**

A qualitative study using semi-structured interviews.

**Setting and participants:**

27 South Asian female doctors and nurses, employed across NHS trusts in London, Greater Manchester and Liverpool, were recruited through purposive snowball sampling between 2021 and 2022.

**Results:**

This study was conducted during the COVID-19 pandemic, a period when existing workplace inequality became more visible and consequential. Although the research was initially motivated by evidence of disproportionate COVID-19 risk among ethnic minority healthcare staff, participants consistently foregrounded experiences of voice, silence and power within the NHS. It was through these accounts, situated in the heightened pressures and uncertainties of the pandemic, that four key themes emerged: (1) how discrimination and ethnic bias suppress voice; (2) fear of retaliation and the consequences of speaking out; (3) internalised cultural norms and the emotional labour of adaptation; and (4) finding voice through experience and action. Participants reported microaggressions, disproportionate disciplinary scrutiny and informal silencing tactics that left them feeling vulnerable and voiceless. For many, cultural expectations around hierarchy and respect inhibited confrontation, even in the face of unfair treatment. Some women engaged in self-reflexive strategies, learning to interpret institutional codes, recalibrating their behaviour or selectively speaking out. For many, this process of adaptation—learning, recalibrating and navigating institutional expectations—was less a path to upholding their agency and more a survival mechanism within a system they perceived as structurally biased. While a few participants described finding ways to speak out and support others through union membership and legal awareness, most described adaptation as emotionally taxing and ineffectual in the face of structural barriers. Silence (eg, withdrawing, transferring departments, leaving their roles altogether) remained the dominant strategy.

**Conclusion:**

COVID-19 did not create these dynamics, but it did expose and intensify pre-existing constraints on voice in the NHS. Drawing on South Asian women’s accounts, this study provides insight into how institutional and cultural dynamics constrain voice and inclusion, particularly under conditions of heightened organisational pressure. We argue that voice is not just a personal capacity but a structural condition that can either reinforce silence or enable change. Our study highlights the need for structural reforms that strengthen psychological safety, ensure clarity around rights and protections and address the persistent gap between inclusion rhetoric and lived experience.

**STRENGTHS AND LIMITATIONS OF THIS STUDY**

- This study provides the first UK qualitative examination of workplace voice and silencing among South Asian women healthcare professionals during the COVID-19 period.
- The study used in-depth semi-structured interviews, enabling detailed exploration of participants’ lived experiences.
- Purposive snowball sampling facilitated recruitment of a group that is often under-represented in National Health Service (NHS) workforce research.
- As a qualitative study, the findings may not be generalisable.
- The study did not include perspectives from NHS managers, employers or human resources staff, limiting methodological triangulation.

## Background

 South Asian healthcare professionals form a critical segment of the National Health Service (NHS) workforce, particularly in hospital and community settings. According to NHS Workforce Ethnicity Data (2022),^1^ Asian staff constitute over 32% of doctors in Hospital and Community Health Services yet occupy only 15% of managerial roles and just 11.3% of senior leadership positions. This stark mismatch between clinical presence and leadership representation points to persistent structural barriers. As shown by data from the Certificate of Completion of Training (CCT) Class of 2023,^2^ many from the growing pipeline of South Asian clinicians report feeling undervalued, scrutinised and marginalised in career advancement. The COVID-19 pandemic is a critical context for exploring these dynamics because it intensified organisational pressures and narrowed opportunities to raise concerns, rendering existing constraints on voice more visible and consequential.

Longstanding structural inequalities within the NHS became more visible and were exacerbated during the COVID-19 pandemic.^3 4^ Ethnic minority healthcare professionals already navigating systemic barriers were disproportionately affected by unsafe working conditions, inadequate access to personal protective equipment (PPE) and inconsistent guidance.^5 6^ Despite raising legitimate concerns, many faced institutional resistance or retaliation for speaking out. Fear of being labelled ‘difficult’ or subjected to disciplinary action further discouraged open dialogue, particularly among staff whose ethnicity attracted heightened scrutiny.^7^,^10^ Rather than serving as a catalyst for reform, the pandemic often reinforced existing power imbalances—penalising those who spoke out and deepening a pervasive culture of silence within the healthcare system.

Compounding these structural barriers is a deeply embedded culture of silence, reinforced by internalised norms around authority and professionalism, particularly among ethnic minority staff. For many South Asian healthcare professionals, cultural expectations that emphasise deference to hierarchy can make it especially difficult to question senior colleagues or challenge institutional practices, even in the face of discrimination or unsafe conditions.^11 12^ For women, these pressures are intensified by gendered expectations and racialised stereotypes, which further suppress voice and heighten the risks of speaking out.^7 13^ Broader concerns about free speech surfaced in reports of NHS staff being threatened or disciplined for raising safety concerns during COVID-19, and for speaking out about unsafe conditions, lack of PPE and systemic failures.^14^ Research has consistently pointed to deep-rooted institutional inertia and structural racism within the NHS, manifesting in heightened job insecurity, disproportionate disciplinary actions and a pervasive fear of reprisals for speaking out.^1 8 9 15^ These pressures were especially acute for ethnic minority professionals who were already contending with a climate of institutional mistrust. Studies suggest that ethnic minority staff are up to 20 times more likely than their white colleagues to face punitive measures for comparable infractions,^15 16^ despite the introduction of the NHS Workforce Race Equality Standard. These barriers emerge early in clinical careers, and they disproportionately affect ethnic minority women through exclusion, bias and reduced voice.^17^ These institutional admissions underscore the fact that racial inequality in the NHS can no longer be viewed as a matter of individual perception; rather, it is a formally recognised, systemic failure.^16 18 19^

Yet, although these disparities are gaining increasing recognition, the specific experiences of South Asian women remain significantly underexplored, in particular how gendered cultural expectations, racialised scrutiny and the lack of institutional support converge to suppress their professional voice.^11^ Existing research^7 14^ highlights that women of colour in clinical settings often face intersecting disadvantages that undermine their capacity to challenge discriminatory practices or unsafe conditions, and when they do so, they may encounter punitive organisational responses.^14^

Understanding how voice is constrained, negotiated or withheld requires attending not only to formal structures, but also to the meanings participants ascribe to speaking out, remaining silent and assessing personal and professional risk, particularly under conditions of heightened uncertainty and risk such as those produced by the COVID-19 pandemic. Recent commentary on the state of the NHS reinforces the urgency of addressing these dynamics. A 2024 report from the Institute for Public Policy Research identifies ‘lack of staff voice’ as a key driver of declining productivity and deteriorating patient care.^12^ The report criticises punitive, top-down reforms as demoralising and counterproductive. It highlights the long-term consequences of silencing frontline workers—particularly those from ethnic minority backgrounds—and shows how failure to engage staff undermines morale, retention and the effective use of public investment, exacerbating the workforce crisis. Against this backdrop, our study explores how 27 South Asian women healthcare professionals managed silence, risk and resistance during the COVID-19 pandemic, a context in which long-standing dynamics of voice and silencing became especially pronounced.

For this reason, we adopted an interpretivist and inductive qualitative approach using in-depth interviews to explore how South Asian women healthcare professionals made sense of their experiences within the NHS during the pandemic. This approach allows participants’ accounts to foreground social, cultural and institutional conditions that shape voice and silence, illuminating how long-standing inequalities were lived and managed in practice.

In doing so, we draw on scholarship that conceptualises voice not just as an individual act, but as a condition shaped by social and institutional structures.^20^,^22^ We shift attention from individual behaviour to the institutional context^23^ and show how symbolic inclusion efforts often mask deeper silencing in hierarchical organisations^24^ where voice acts as a barometer of workplace equity and power distribution.^25^ These insights inform our approach and reinforce why voice—and its suppression—must be central to any analysis of institutional inequality in the NHS.

Against this broader policy and institutional scenario, we asked: how did South Asian women healthcare professionals experience and navigate voice, silence and risk within the NHS during the COVID-19 pandemic? We use COVID-19 as a revelatory context to explore how constraints on voice, already present in everyday working life, become especially consequential under heightened organisational pressure. The study foregrounds South Asian women’s accounts of speaking out, being dismissed and feeling unable to refuse unsafe or unfair working arrangements.

## Methods

A qualitative, interpretivist and inductive approach was adopted to explore the lived experiences of ethnic minority doctors and nurses in the NHS during the COVID-19 pandemic. The study aimed to examine how management practices, cultural expectations and professional hierarchies shaped their ability to voice concerns. A constructivist and qualitative methodology was well-suited for this research, as it allowed participants to articulate their perspectives while enabling the researcher to build the trust and rapport essential for eliciting rich, meaningful data.^26^

### Participant selection and recruitment

Participants were eligible if they were women who self-identified as Pakistani, Bangladeshi or Indian and were employed as doctors or nurses in clinical roles within the NHS during the COVID-19 pandemic. Using purposive sampling, we recruited South Asian women across these groups to ensure variation in seniority, age and years of experience, which enabled data saturation through the collection of in-depth, layered and nuanced data.^27^ Sample size was not prespecified but emerged through recruitment conducted over approximately 12 months during the COVID-19 pandemic, when participation availability was variable. Researchers collaborated with gatekeepers within each NHS trust to identify potential participants. The invitation to participate was distributed by the trust via internal communication channels and circulated to members of ethnic minority staff networks. These communications included a summary of the study’s aims, assurances of confidentiality and instructions on how to express interest. Given the identifiable nature of some professional contexts, we have protected participant anonymity by not providing in this article a breakdown of staff categories by role, department or trust. Given the potential sensitivity of discussing workplace discrimination and silencing, ethical consideration was given to the possibility of emotional distress, and participants were able to pause, decline to answer questions or stop the interview if needed.

### Data collection

Prospective participants made initial contact with the first author via email to confirm their willingness to participate. During this interaction, we provided detailed information about the study, including its objectives, the nature of their involvement and the measures in place to protect their anonymity. Interviews were only scheduled once participants had voluntarily agreed to take part with a full understanding of how their data would be used. Our primary goal was to create a platform where participants felt heard, respected and safe in sharing their perspectives. Prior to the interviews, we explained the consent process, reassured participants about confidentiality and emphasised the voluntary nature of their involvement to ensure they were comfortable discussing their experiences. We also committed to using pseudonyms in all research outputs to maintain anonymity. Participants were informed that interviews would be conducted online, recorded for transcription purposes and securely stored in the University Research Data Storage Unit for up to 5 years. They were made aware that by attending, they were agreeing to the recording of their contribution, and that these recordings would only be accessible to the research team.

On the day of the interview, participants reviewed the information sheet again and were given the opportunity to ask questions and raise any concerns. Verbal informed consent was obtained at the start of the interview, and participants were reminded that they could withdraw at any time during or after the interview without consequence but that their responses up until the point of withdrawal would be retained. No participants withdrew during or after the interviews, though one individual chose not to participate before the scheduled interview due to personal reasons.

All interviews were conducted online due to COVID-19 restrictions and the time constraints faced by participants during the pandemic. The interview guide was developed based on existing research and news reports on the experiences of ethnic minority healthcare staff in the UK during the pandemic (see online supplemental appendix 1). The guide was designed to explore several key topics to understand the multifaceted experiences of South Asian professional women in the NHS during this period. Specifically, it examined changes in household and family dynamics, daily routines and socioeconomic circumstances, as well as how self-identification and cultural influences shaped their professional trajectories. Additionally, it addressed workplace experiences, including interactions with colleagues and patients, and instances of discrimination, assessing their impact on participants’ ability to voice concerns. It also explored the broader effects of the pandemic on personal and professional aspects of the participants’ lives, highlighting the interplay between structural inequalities, cultural conditioning and institutional responses. Instead of predefining issues (eg, workplace discrimination) as central to our analysis, our approach allowed participants to freely discuss the aspects of their experiences they found most significant, such as the intersection of cultural identity, family responsibilities and career development (table 1).

**Table 1:** Topic guide: the socioeconomic impact of COVID-19 on South Asian women in the NHS

Topic area	Key areas explored
Impact of COVID-19 on work and daily life	Changes in work responsibilities and job security; balancing work, household and caregiving duties; emotional and mental well-being during COVID-19
Workplace challenges and support	Career progression and barriers in the NHS; experiences of discrimination or bias; workplace policies and support systems during COVID-19
Financial and economic impact	Changes in income, savings and financial stability; additional financial responsibilities due to COVID-19; access to financial or employment support
Health and safety in the NHS	Workplace exposure and PPE availability; effectiveness of COVID-19 policies and response; personal risk perception and health concerns
Community and social factors	Role of family and community support; social isolation and coping mechanisms; perceptions of COVID-19 and vaccination
Lessons and recommendations	Key challenges and takeaways from the pandemic; improvements needed in NHS policies and workplace support; recommendations for future crisis preparedness

NHS, National Health Service; PPE, personal protective equipment.

### Data Analysis

All interviews were transcribed verbatim and thoroughly checked for accuracy by the research team. Data were analysed using an inductive thematic analysis.^28^ The first two authors independently read and re-read transcripts to familiarise themselves with the data, and then manually coded an initial subset of transcripts, line-by-line, using a coding template based on the interview guide. Intercoder reliability was established. This coding approach was then applied to the remaining transcripts, with ongoing refinement as analysis progressed. Codes were compared, collapsed and organised to identify patterns, relationships and higher-order themes and subthemes. The coding authors reviewed and discussed the transcripts to reach a consensus on emerging themes and subthemes. Reflexive memos and regular peer-debrief meetings examined how the researchers’ positions shaped coding decisions and the development of themes. This approach enabled structured identification of key themes while allowing insights to emerge from the data. Consideration was given to researchers’ ability to manage and mitigate any unforeseen discomfort or distress during the interviews. To ensure ethical and professional standards, the research team underwent Good Clinical Practice training prior to conducting interviews. The first author’s South Asian background and deep connection to the studied communities provided invaluable insight into the research process and data interpretation, allowing nuanced understanding of the subtle discrimination participants encountered. Having already navigated both personal and professional spaces within this cultural framework, the first author had developed a keen awareness of the persistent, tacit inequalities faced by South Asian women in their careers. In contrast, the second author brought an objective perspective, analysing the data through an anthropological lens. Acknowledging the first author’s insider position, we took deliberate steps to ensure that personal biases did not influence our interpretation. The second author’s external standpoint complemented the first author’s insider perspective, offering a balanced and enriched analysis that strengthened our theoretical insights. Building on our positionality as researchers, we were acutely aware of our role as listeners to these often-silenced voices. Throughout this work, we were guided by a commitment to surface not only the visible structures of discrimination but also the quieter, everyday exclusions that shape professional life. In bearing witness to these narratives, we recognise our responsibility to honour the emotional labour of our participants and to contribute to a broader conversation about equity and belonging in the NHS.

We used the Standards for Reporting Qualitative Research (SRQR) reporting guideline to draft this manuscript, and the SRQR reporting checklist when editing (see online supplemental file A). We also completed the COREQ (Consolidated criteria for Reporting Qualitative research) checklist (see online supplemental file B).

As it is the nature of qualitative study, outcomes were understood as conceptual rather than in statistical terms. The study focused on participants’ experiences of voice and silence within the NHS, particularly in relation to raising concerns about risk, safety and fairness. We examined participants’ description of adapting to, coping with or resisting situations in which voice felt constrained. The COVID-19 pandemic was a context in which heightened organisational pressure rendered the significance and consequences of voice and silence more visible.

### Patient and public involvement

Neither patients nor the public were involved in the design, conduct, reporting and dissemination of this research.

## Results

### Demographic data

We employed a purposive snowball sampling strategy to recruit 27 professional women from Pakistani, Bangladeshi and Indian communities in key UK metropolitan areas: London, Greater Manchester and Liverpool. All participants were employed in clinical roles, including consultants and nurses, within the NHS during the COVID-19 pandemic and shared experiences shaped by their ethnic and professional identities. Participants varied in age, seniority and years of experience within the NHS, offering a broad perspective (see table 2).

**Table 2:** Participants’ demographic information

Grade	
Nurse Band 3	1
Nurse Band 5	4
Nurse Band 6	6
Nurse Band 7	3
Consultant	13
Education	
College	4
University	23
Religion	
Hindu	9
Muslim	12
Christian	5
No religion	1
Ethnicity	
Indian	14
Pakistani	10
Bangladeshi	2
Age	
55–64	8
45–54	12
35–44	6
25–34	1
Birthplace	
India	12
Pakistan	3
Middle East	1
UK	11

Interviews were conducted in English by both researchers and lasted between 90 and 110 min. In total, we analysed approximately 41 hours of interview data, amounting to 1245 pages of transcripts. The themes and insights generated through our analysis informed the development of a set of practice-based recommendations, which are summarised in table 3.

**Table 3:** Recommendations for practice

Domain	Recommendation
Psychological safety and voice	NHS trusts should provide dedicated, culturally sensitive induction programmes that explain staff rights, protections and expectations from day one. Senior leaders and line managers must undergo training on bias, power dynamics and voice suppression, with clear accountability structures. Create anonymous, independent reporting mechanisms co-designed with minoritised staff to increase trust in institutional safeguards.
Communication and cultural norms	Include training on different communication norms and workplace behaviours to reduce penalisation of culturally divergent interaction styles. Implement reverse mentoring or shadowing schemes pairing senior leaders with minoritised staff to foster understanding and reduce top-down stereotyping.
Structural reforms and accessibility	Make staff networks, union information and grievance procedures widely visible, accessible and clearly explained, especially to newly recruited staff. Translate key HR documents and guidance into multiple languages and formats to improve accessibility and reduce misunderstanding or fear.
Support and retention	Develop informal peer support structures within departments, enabling staff to share concerns and navigate challenges before formal escalation. Publicly showcase examples of staff advocacy, success in resolving grievances or leadership by minoritised professionals to build confidence in institutional responsiveness.

HR, human resources; NHS, National Health Service.

### Emerging themes

**Figure 1:**
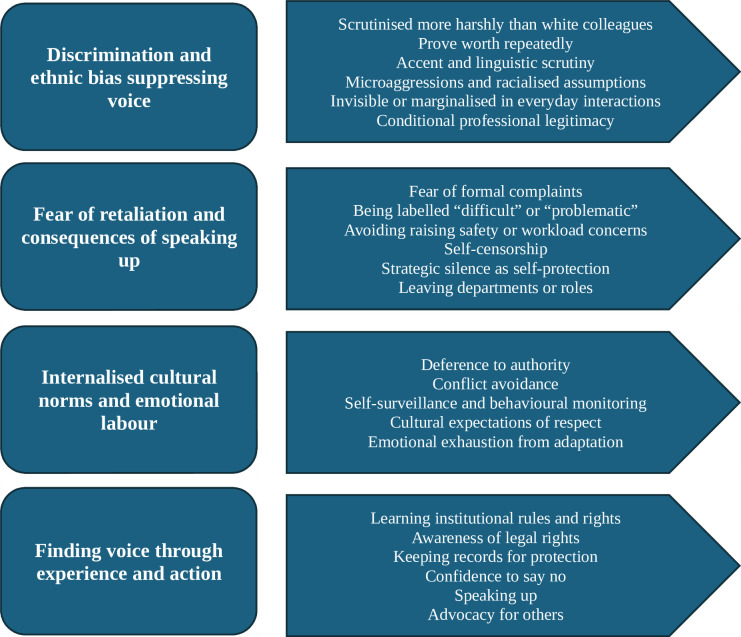
Themes and subthemes identified from the data analysis.

Four primary thematic domains emerged from the data: (1) how discrimination and ethnic bias suppress voice, (2) fear of retaliation and the consequences of speaking out, (3) internalised cultural norms and the emotional labour of cultural adaptation and (4) finding voice through experience and action (figure 1).

### How discrimination and ethnic bias suppress voice

Participants described how discrimination and ethnic bias curtailed their ability to speak up in the workplace. Their accounts related both formal mechanisms (eg, disproportionate disciplinary action) and informal, everyday processes like social exclusion, linguistic scrutiny and assumptions of incompetence. Many reported being penalised more harshly than their white British colleagues for comparable behaviours. Some avoided raising concerns altogether due to fear of isolation or reputational damage. Others described the pressure to perform flawlessly, linking professional legitimacy to perfection, linguistic conformity and the endorsement of white colleagues. In this context, silence was not imposed through overt prohibition; rather, it was produced through cumulative and embedded patterns of marginalisation.

One consultant explained:

Ethnic minorities are 20 times more persecuted than their English counterparts. It’s the GMC [General Medical Council] report … anything you say is undermined. So you can’t speak. If you do, you’re called derogatory, and then the whole institution stands against you. (C13)

The disproportional number of referrals—specifically, formal disciplinary referrals to the GMC—received by South Asian professionals creates a climate in which speaking out is perceived as risky.

Participants also described diffuse mechanisms of silencing, which were rooted in the fear of exclusion or not belonging. Some reported subtle forms of social distancing or professional sidelining that reinforced their marginality. One nurse recounted:

What makes me feel uncomfortable is to think that other people might be uncomfortable with me. For example, I notice people hesitating to sit next to me at the canteen … they removed me from patient interactions, something that is at the core of my job, I felt invisible. (N1)

Such experiences of social erasure, while not formally disciplinary, contributed to a wider climate in which participants felt their presence was unwelcome or conditional. These processes operated in parallel with more explicit risks, generating a layered experience of silencing.

Participants also spoke of a persistent pressure to meet higher standards to secure recognition or avoid criticism:

It would appear that for them to respect a person who is non-white you had to be outstanding … you were not allowed to make a mistake. (N3)

This demand for perfection was compounded by linguistic scrutiny. Respondents, regardless of whether they were UK-born or internationally trained, felt their speech, accent and communication style were subject to disproportionate evaluation, which they experienced as undermining credibility and confidence to speak:

Women are discriminated against more because they have kids, but for Asian women … also speaking properly or speaking well can be an issue at work if you are Asian. (N5)

Despite being qualified and experienced, I earn less than white people … it’s my accent, I think. (C7)

I don’t always feel comfortable with the way I speak … I need to be more succinct. (C11)

Participants also described a persistent need to earn credibility in the workplace regardless of their experience or seniority. For some, even long-term service did not shield them from scrutiny; professional legitimacy had to be continuously re-established, particularly with newer colleagues. This ongoing pressure to gain validation from white British colleagues functioned as a gatekeeping mechanism. Respect was not assumed; it had to be continually earned, making inclusion conditional and precarious:

As a member of an ethnic minority, I need to constantly prove myself to my colleagues. While my long-term colleagues recognise and swear by my abilities, newcomers or those unfamiliar with my work often scrutinise me more harshly. (C13)

These judgments can extend to other aspects of my work environment. If I am not respected by my English colleagues, others, including nurses and senior staff, are more likely to treat me unfairly or disrespectfully too. This lack of respect can have cascading effects, leading junior staff to also disregard me. (C13)

In addition to formal and interpersonal scrutiny, many participants reported microaggressions, comments, jokes or assumptions. Although these were often framed as casual, they cumulatively reinforced feelings of exclusion. One consultant recalled:

When I was elected to an important leadership role, the first day, the chief exec, a white woman, says to me: ‘I’ve heard that Indian women become doctors to get good husbands …’ and I was sitting there thinking, shall I walk out and make a massive statement and explode? Or do I stay and change it? … I stayed. (C14)

Even respondents with positional power often chose silence, not out of acquiescence but as a strategic choice to preserve influence. In other cases, the nature of exclusion was more ambiguous, leaving participants uncertain about whether what they had experienced could be termed racism at all:

Overt racism is not there. But I would say that I’ve been treated in a way that I thought if I was a white middle-aged man, this wouldn’t have happened. It is that kind of racism that is hard to put your finger on and call out and say it is racist. (C12)

This ambiguity generated self-doubt and hesitation. Rather than risking dismissal or being labelled oversensitive, participants often chose silence. In this context, the act of speaking out was not merely difficult—it was shaped by layers of calculation, risk and emotional strain.

### Fear of retaliation and the consequences of speaking out

Fear of retaliation emerged as a pervasive theme across departments and roles. Whether they were raising concerns about patient care or questioning workplace dynamics, participants described a culture in which the risks of expressing dissent left them feeling that they did not “have a voice at all“.

Highlighting any kind of discrepancies or discussing patient care concerns can lead to being labelled as problematic or having behavioural issues. It makes me feel that as an ethnic minority, I have no voice and am unable to express concerns without facing repercussions. (C13)

Participants explained that not feeling heard undermined their confidence to raise issues, especially those related to patient safety or institutional inequality. This voicelessness was particularly acute when addressing unequal workloads. Multiple respondents described stark workload disparities, including accounts of seeing nearly five times as many patients as their white colleagues during equivalent shifts.

The white consultant sees five patients in the whole month compared to an ethnic minority who will see 29 … and if you say that, you are being rude. You are undermining … You can’t explain that. You can’t even say that. If you say that … then you will undermine, and you will be subjected to a medical regulatory body … No voice at all. (C12)

I work in a busy, busy ward where often patient are admitted with multiple problems, and I look after their cases now as I see most of the sick patients and most of the new patients … that four hours I’ve been given to do this … my colleague will see a maximum of six to seven patients in that time. (C14)

These accounts reflect a compounded fear where reputational risk is closely tied to the threat of regulatory action. Speaking out was perceived not as a protected right but as a dangerous act that could trigger a formal investigation, regardless of intent. Within this context, silence often became a strategy of self-protection:

I try not to interrupt too much for fear of being undermined or patronised. So mostly I interact with the nurses and the junior doctors, and I try not to interrupt so much with my colleagues because there’s a lot of undermining, condescending, patronising. (C13)

There is nothing to be gained from speaking out … I like to deny that’s what’s happening. (N1)

Participants described carefully navigating interactions in order to reduce the risk of being singled out or misinterpreted. This sense of hopelessness—combined with a fear of reprisals—meant that silence, selective engagement or denial often replaced formal reporting or open discussion.

Mostly, though, I ignore most things because I know nothing will be good if I complain. (N5)

I would never ever make a complaint or go to HR ever. I would rather leave a place. (N20)

For some, strategically exiting a post was seen as safer than using formal report mechanisms, which were widely viewed as ineffective or even personally damaging. These decisions—whether to leave, stay silent or endure—were not isolated but shaped by a wider organisational culture that discouraged dissent and rewarded compliance, even in the face of discrimination. Participants consistently described how raising concerns, whether about workloads, patient care or interpersonal mistreatment, met with the threat of disciplinary action or subtle exclusion. As such, formal actions for inclusion and messages around well-being became misaligned with day-to-day experiences. When openness was promoted rhetorically but speaking out felt risky in practice, many chose silence as a safer option.

This climate of fear shaped not only what participants said, but also how they carried themselves in daily interactions. All participants expressed some degree of concern about how they presented themselves in the workplace—how they spoke, wrote and were perceived. These anxieties extended beyond formal communication into everyday encounters, prompting many to limit contact with certain colleagues to avoid situations where they might feel diminished or dismissed. One participant offered a striking reflection on how these anxieties intersected with identity and informal power dynamics:

There is a pecking order: White women still have a really quite hard time in medicine … probably just not as hard as someone like me … BUT I don’t have as hard a time as someone who might wear a hijab and work. So, there is just this pecking order. If you are a white man, usually OK. If you’re a white woman, you can have some problems. If you’re a non-white woman, etc. And then, you know, if you’re a Black woman, you’ve got the worst experience of all. (C13)

Such reflections reveal a deeply embedded awareness of informal hierarchies in clinical spaces. Participants described navigating these hierarchies through continuous self-monitoring and adaptation—strategies used to maintain professional safety in an environment where voice is often constrained by fear.

### Internalised cultural norms and the emotional labour of cultural adaptation

In the face of silencing and exclusion, self-reflection became a complex and often ambivalent response. Several professionals reflected not only on their present behaviour, but also on deeply rooted familial and cultural conditioning. Their internal (and frequently unfavourable) comparisons with white British colleagues sometimes led to feelings of self-blame or inadequacy. One consultant, for example, described how she was consciously raising her son to speak more openly and critically than she had been allowed to:

We were brought up in a way to listen to our elders or our seniors. You listen. Even if we know that’s wrong, we were not allowed to question … So now I encourage my son to speak up. (C12)

While some expressed a desire to break these patterns for the next generation, others described the ongoing difficulty of asserting themselves at work. One nurse noted how long it took to feel entitled to speak up ‘like the white people’. For others, such directness felt unfamiliar or inappropriate—at odds with the values they had grown up with.

This hesitation was not framed as simple shyness but as something more deeply rooted. Cultural norms that emphasised respect, humility or restraint shaped how participants responded to injustice and conflict. One nurse shared how she had been taught to interpret others’ actions generously, even in the face of harm:

My dad has always instilled in me, you always give people the benefit of the doubt, like there might be something else going on in someone else’s life … Maybe someone is having a bad day. (N1)

For some, this mindset acted as a form of emotional buffering—a way to navigate microaggressions without confrontation. Others described a more general preference for handling issues privately, without escalation:

In my cultural background, there’s an inclination to handle problems internally. (N8)

This approach to conflict was widely shared and often seen as a source of personal strength. But it carried a cost. Quiet endurance fostered resilience, but it could also lead to missed opportunities, unacknowledged workloads and a growing sense of invisibility within the institution.

Some participants described how, over time, they began to challenge these patterns. One consultant spoke about the need to adopt a different communication style in order to progress professionally:

White people say no, so she had to learn that too: White will just say ‘That’s not right.’ But as we are, should I say this? It’s, you know, what will they think about us? But then over the years, through my experience working with people in this culture, I realised that’s not going to work. That’s not going to push me forward in my career. (C12)

She went on to describe this shift not as a simple behavioural adjustment, but as a deeper emotional and psychological transformation:

Unlearning a lot of things from the way I’ve been brought up and learning new things as well. That is for my survival. That is for me to grow, go higher, kind of. (C12)

Her account highlights the ongoing negotiation between inherited cultural values and the behavioural expectations of the workplace. For many, adaptation involved walking a difficult line: retaining their dignity while learning to function in a system that often rewarded assertive self-promotion and penalised deference. Adaptation was both emotionally demanding and framed as necessary for survival and progression. Speaking out was experienced as professionally risky and personally difficult, given long-standing implicit norms around deference and restraint.

### Finding voice through experience and action

Faced with daily experiences of discrimination, ethnic bias and microaggressions, participants described a spectrum of survival strategies ranging from quiet perseverance to institutional confrontation. A recurring theme was the role their work ethic and professional pride played in helping them endure and persevere.

Despite the challenges, I am proud of my work … (C7)

Professional pride and ethical integrity served as internal resources—sources of strength that helped sustain participants through daily repeated adversity. For some, resilience was described as a form of moral strength; for others, it was tied to merit and performance, with success functioning as a silent rebuttal to bias.

I am determined to be resilient … I try not to take these comments to heart. (N8)

For that participant, resilience was both a protective shield and a strategic response; she consciously chose to focus on her values rather than internalise the harm. For others, perseverance and achievement were seen as the clearest form of resistance. Voice is not expressed through open dissent, but reclaimed through persistence, competence and visible success:

I just keep going … and I proved myself a lot to get where I am. (C10)

Some participants found that maintaining a strong work ethic and ’keeping their head down’ was a viable strategy, even if it was often accompanied by feelings of frustration or resentment. Others turned to more relational forms of expression such as mentoring junior colleagues or speaking with trusted peers. A few engaged with formal channels, including union membership or written reports. One consultant described how she had responded to a difficult relationship with a colleague by formally lodging a report:

I always ensure [to] keep record of everything as evidence. (C7)

Here, record-keeping became an act of resistance, transforming vulnerability into preparedness and helping her navigate the process from a position of control. For others, taking action took more time. Another consultant described having left her department after persistent undermining from management. Eventually, she chose to address the situation:

I did confront him a few years down the line. (C16)

In this case, delayed confrontation was a form of retrospective assertion. Across participants, voice did not simply emerge—it was pieced together over time through reflection, timing and necessity.

In some cases, participants took more immediate and assertive action. One consultant described how a workplace conflict prompted her to engage with the union and learn more about her rights:

I already had a big case against my manager, and I was in touch with the union. I also came to know more about the laws and more about my rights. So, I realised, previously I would have been afraid to say no to my manager. But I was very confident that I could say no, and nobody could harm me or my job. (C12)

Here, institutional knowledge became a source of power, replacing fear with confidence and reorienting the participant’s relationship to authority. The consultant then reflected on how this experience transformed her understanding of institutional support:

I’m still there with the union because I realised what all the powers they can give you, what all the support they can give you, and how there are lots of rules which you do not know. You come and do your job and go, but only when you encounter a problem do you realise lots of things.

Her account highlights that the shift from silence to action was made possible by experience and access to information. Only two participants in our study had joined the union, and only after major difficulties at work had prompted them to find out what protections existed. This highlights a serious issue: minoritised individuals may delay seeking support not because the support is not needed but because they have limited awareness of their rights or are concerned about potential backlash.

Having found her voice, the same consultant began advising and supporting others, particularly newcomers who might be unfamiliar with their rights or hesitant to speak out:

Today, if I see anybody suffering mistreatment or discrimination, I’ll tell them: You don’t have to suffer in silence. You have your rights … So, anybody new joining in and whom I know, I tell them: There will be people like this, that is quite common. You should not have to suffer.

Her advocacy reflects a broader sense of responsibility. What began as a personal struggle evolved into a form of protective solidarity, extending even to colleagues outside her ethnic group. Her ongoing outspokenness is not just about redressing her past experience, but to prevent future harm to others:

Why I still complain and make a fuss about it is because I have experience, and it’s not a good experience. And I do not want even any other Caucasian under him to suffer.

This kind of advocacy was common among senior consultants, particularly those who perceived nurses and junior doctors as having even less voice than themselves.

## Discussion

To our knowledge, this is the first study to explore how South Asian women doctors and nurses navigated voice, risk and institutional silence within the NHS during the COVID-19 pandemic. The study was part of a wider investigation into the experiences of minoritised healthcare staff, with a specific focus on how participants responded to structural inequalities in their everyday working environments. Participants described a range of barriers to raising concerns, including fear of retaliation, cultural expectations around deference and institutional mistrust. Research shows that ethnic minority doctors in the NHS are twice as likely as white doctors to be reported to the GMC by their employer, rising to 2.5 times for those trained abroad.^29^ While mechanisms such as unions, staff networks and formal report procedures were present, participants viewed these as unevenly accessible and often unsafe to use.

Drawing on theories of institutional voice,^21 22^ we understand ‘voice’ as not simply the presence of formal mechanisms for expression, but as a contextually embedded practice shaped by power relations, cultural norms, identity and perceptions of safety. Institutional engagement is not only procedural; it is embodied, shaped by intersectionality and the implicit norms that govern whose voices are legitimised.^30 31^ In the wider ‘experience economy’, organisations often position inclusion as a marketable asset.^32^ But when structural inequalities remain unaddressed, these promises offer little real protection. This framing helps illuminate how formal channels often fail to account for the lived realities of those expected to use them. Our findings suggest that even when procedural channels exist, they are frequently experienced as exclusionary or symbolic.

We identify four key patterns that shape this dynamic: silencing and risk perception; intersectional disadvantage and occupational scrutiny; cultural adaptation and behavioural constraint; and institutional dissonance and symbolic inclusion. Yet by learning how to navigate institutional processes, joining unions or supporting others, some participants were able to take action within this constrained landscape in ways that shifted their own relationship with risk and influenced how others around them understood what was possible. These acts, while often cautious and costly, became forms of advocacy that disrupted expectations and, at times, inspired or unsettled colleagues. Rather than signalling resolution, they reflect an ongoing negotiation between silence and voice—one shaped by the ability to strategically navigate structures that are often exclusionary by design.

### Silencing and risk perception

This study found that the risk of speaking out was a defining feature of participants’ working lives. Previous research has shown that minoritised staff in the NHS are less likely to use formal reporting routes, often due to fear of reprisal or a lack of trust in outcomes.^1 15^ Our findings confirm and extend this, showing how institutional mistrust is not just reactive, but anticipatory—shaped by cumulative experiences of marginalisation and observation of how others are treated.

The perception of risk was often internalised, influenced by cultural norms of deference and a reluctance to challenge authority. Participants described adopting self-monitoring behaviours to minimise attention and avoid conflict. These strategies mirror findings from previous studies documenting how staff from ethnically minoritised backgrounds engage in ‘institutional passing’, adapting their behaviour to fit dominant workplace norms in order to avoid sanction.^31^ While many participants described how silencing takes shape through differential disciplinary actions, the requirement to overperform for recognition and persistent microaggressions, they also identified ways in which they navigated these situations to retain their jobs and even thrive. Adaptation, positive self-talk, the pursuit of excellence, legal awareness, solidarity with colleagues and union support were some of the key tools for resisting marginalisation.

While some participants eventually engaged with formal structures, this was rarely proactive. Legal knowledge, record-keeping and union support were typically accessed only after prolonged exposure to harm, suggesting that confidence in institutional safeguards remains low. This reflects broader concerns about symbolic inclusion in public sector organisations, where diversity mechanisms may exist more for reputational assurance than to offer meaningful protection.^21 33^ These strategies were taxing for the participants and while some described moments of strength or clarity, for others, it felt like their only option was to leave. What emerged from the interviews was a constant effort to navigate hostile systems, challenge norms and find ways to stay in the profession under difficult conditions.

These findings reinforce the argument that silence should not be interpreted as disengagement or consent, but as a calculated response to a workplace context perceived as risky. It has been argued that voice is shaped less by formal opportunity than by perceptions of psychological safety.^21^ For South Asian women, this safety was often absent, being replaced with careful calculation, restraint and, in many cases, withdrawal. Interventions that rely solely on procedural access to voice are therefore insufficient unless attention is given to the social, emotional and cultural conditions that underpin its use. Some participants spoke of a shift in their own identity at work: from passive observers to advocates not only for themselves, but also for others facing similar challenges. These moments of transformation suggest that although institutional and cultural barriers routinely suppress ethnic minority voices, some participants were able to carve out space for agency in a system of constraints through persistence, networks and hard-won awareness. These institutional and cultural barriers demanded additional labour—emotional, strategic and relational—from South Asian professionals seeking to be heard.

### Intersectional disadvantage and occupational scrutiny

South Asian women in the NHS experience multiple and intersecting layers of silencing, some of which are hard to pinpoint but which include heightened professional scrutiny, workload disparities, social exclusion and persistent microaggressions. Participants described the constant labour required not only to meet high performance standards but also to navigate informal hierarchies that determine whose voices are heard and whose expertise is validated. This process of validation was not only racialised and gendered but also linguistic—participants’ accounts resonate with scholarship on accent bias and epistemic injustice.^34^ Bias operates not only structurally, but also psychologically through internalised self-surveillance (a form of constant self-monitoring shaped by awareness of racialised expectations). This is connected to cultural bias, which often surfaces through jokes or a curiosity that masks implicit judgements. Credibility is thus often unevenly distributed based on speech patterns, pronunciation and perceived ‘professional’ voice. In this context, accent functioned as a marker through which clinical authority was subtly questioned, requiring South Asian women to engage in additional labour to have their knowledge recognised and legitimated.

Many shared that professional recognition was often contingent on the endorsement of white colleagues. Even after achieving senior roles, participants reported having to repeatedly re-establish their credibility in the eyes of new colleagues or managers. These findings align with research on intersectional discrimination that highlights how racism and sexism combine to impede the career progression of ethnic minority women, often confining them to lower-level roles in a form of occupational segregation.^35^,^39^ This ‘double jeopardy’ reinforces systemic inequality and limits access to leadership, autonomy and voice. Our study confirms that South Asian women in the NHS face not only individual biases but also structural barriers to advancement. For this reason, we argue that intersectionality must be central to both research and NHS workforce policy. A truly effective approach must go beyond addressing overt and covert discrimination to include reforms of organisational structures that perpetuate inequality. Without these changes, the burden of navigating inequality continues to fall on individuals rather than institutions. These experiences suggest that bias operates not only through overt exclusion, but through ambient forms of marginalisation that shape who is seen, heard and believed, which aligns with earlier work on intersectionality in public sector employment.^40^

Transparent, trusted policies around job planning, concerns and conflict resolution are essential to level the field. When these systems are clearly communicated and consistently applied, they allow professionals to safely speak up and challenge structural disadvantage. In contrast, symbolic forms of inclusion, unbacked by real structural accountability, offer little more than reputational cover and risk abrogating the emotional and strategic labour of equity work to ethnic minority professionals alone.

Microaggressions were described as a particularly persistent form of low-level harm that reinforced social exclusion. Participants recalled incidents that, seemingly trivial in isolation, had cumulative effects on psychological safety and professional confidence. Some referred to offhand comments about nationality or skin colour; others described how their clinical judgements were second-guessed or dismissed. Several said they chose to remain silent after witnessing how others were treated for raising concerns, reflecting patterns of vicarious silencing. The tendency to withhold voice was not due to a lack of grievance; rather, it was a calculated response to risk, exhaustion and prior experience.

The COVID-19 pandemic intensified these dynamics, particularly for staff already navigating marginalised identities. Participants described increased institutional pressure to perform even as resources became scarcer and support more limited. Speaking out about safety, PPE or emotional distress was discouraged or penalised. Silence became, for some, the only viable strategy. At the same time, the pandemic functioned as a catalyst. It exposed institutional fragilities and brought workplace injustices into sharper focus. For some participants, this prompted greater engagement with unions, legal protections and support networks. The extreme conditions of the crisis thus revealed both the fragility and the potential of institutional voice within the NHS.

The findings point to the limits of symbolic inclusion policies when these are not backed up with structural accountability. While equality statements and staff networks are widely used to promote NHS diversity efforts, participants consistently described these mechanisms as performative. Their accounts are consistent with critiques of diversity policy as mechanisms for reputation management rather than meaningful interventions.^24 33^ In practice, such systems often left staff to navigate inequality on their own.

Improving institutional responses to intersectional disadvantage requires more than expanding access to reports procedures or mentoring schemes. New staff need explicit and early communication about their rights, the institutional processes and what support is available. As shown in this study and others,^18^ the confidence to speak up is more likely to be sustained when individuals have clear pathways and visible examples of accountability, and are supported by leadership that understands the emotional and cultural risks associated with raising concerns. When such structures are in place, they do not just protect individuals—they activate voice, rebuild trust and lay the foundation for collective change from within. To make this real, organisations must back policy with practice through early orientation, visible accountability, mentoring and regular spaces for dialogue that make inclusion tangible, not just aspirational.

### Cultural adaptation and behavioural constraint

Participants’ accounts indicated that voice within the NHS is shaped not only by structural access to mechanisms like trade unions and staff networks, but also by internalised cultural norms and personal histories. Many described a reluctance to speak up not because they lacked opportunity, but because they were uncertain about how their concerns would be received. This hesitation reflects prior research showing that voice is contingent on institutional trust and psychological safety, not merely procedural access.^21^ Even where formal channels were available, participants weighed the emotional cost and potential consequences of using them and often chose silence over confrontation.

These patterns were especially pronounced among South Asian staff, for whom the challenge of speaking out was shaped by both structural barriers and internalised norms. Many participants reflected on upbringings marked by early lessons in deference to authority and the avoidance of conflict, which made it difficult to challenge senior colleagues or speak openly in the workplace even in circumstances where doing so was warranted. The intersection of cultural background and workplace hierarchy often led to second-guessing and self-censorship. Some explicitly compared themselves with white British colleagues, describing assertiveness as something that different expectations of communication and behaviour made less readily available.

Participants’ accounts of consciously ‘unlearning’ inherited behavioural norms and adopting more asserting communication styles point to the deeply embodied nature of voice in professional settings. Practices are not simply created cognitively; they are enacted through bodies shaped by prior socialisation. In this sense, voice is about not only knowing what to say but how to act in relation to power.^41^ Employee experience is never neutral^30^ but formed through cultural memory, identity and bodily presence. These findings suggest that speaking out is dictated by historically and culturally sedimented expectations.

For South Asian women in clinical NHS roles, this helps explain why voice was experienced as both professionally risky and emotionally demanding. Speaking out required participants to confront entrenched norms of deference and restraint, making adaptation more than a matter of skill development. Rather, it functioned as a strategy of survival within organisational contexts, where dominant behavioural norms have not been designed with them in mind. While this process could be experienced as enabling greater professional legitimacy, it was also associated with significant emotional labour. This kind of strategic adaptation or ‘flexing’ often involves suppressing aspects of one’s identity or emotional response in order to align with dominant norms.^31^ Participants’ accounts resonate with this tension, illustrating how the pursuit of credibility through adaptation was accompanied by fatigue, discomfort and a sense of personal cost. Taken together, these findings extend existing work on workplace voice by showing how embodied, culturally mediated forms of adaptation can simultaneously enable participation and reproduce inequality.

Notably, our findings suggest that even when formal mechanisms exist, their effectiveness must be understood within a broader context of socialisation and cultural adaptation. Voice is not merely a procedural issue; it is a deeply personal one, shaped by individual histories and the risks associated with speaking out.^20^ Many participants navigated what they perceived as an informal ‘pecking order’ in which they had to work harder than their white colleagues to gain equal recognition. Cornelius *et al* argue that employee experience is not a fixed benefit that employers can simply offer, but a condition that emerges through everyday interactions shaped by power.^30^ The relentless scrutiny our participants described, combined with the continual need for validation, embeds inequality into the very practice of work. Silencing operates not only through a fear of being penalised, but also through the emotional and professional exhaustion that comes from continually having to justify one’s place. This dynamic points to what can be understood as a continuous negotiation of respect: an ongoing, effortful process through which participants calibrated tone, assertiveness and self-presentation in order to secure professional legitimacy. Respect was neither assumed nor stable; it had to be repeatedly earned and maintained through behavioural adjustment, emotional regulation and strategic silence. This awareness significantly influenced whether, when and how they felt able to raise concerns.

These findings suggest that efforts to improve institutional voice must consider more than formal procedures. They must also address how voice is shaped by culture, emotional risk and interpersonal dynamics. Managers and institutions need to recognise the diversity of communication styles and the psychological burden associated with asserting oneself in unfamiliar or unwelcoming environments. Induction processes and leadership development should move beyond basic compliance to actively foster trust, encourage open dialogue and normalise diverse ways of expressing concern. Without this shift, formal mechanisms risk being perceived as inaccessible or ineffective, especially by those who have historically been marginalised. In short, institutional initiatives that encourage speaking out may continue to have limited impact if they do not address the embodied and emotional dimensions of voice.

### Institutional dissonance and symbolic inclusion

Participants described a stark disconnect between institutional messaging around inclusion and their lived experience within NHS settings. While diversity policies, staff networks and organisational values around equality were prominently promoted, these initiatives were widely perceived as symbolic. Rather than providing protection or voice, they were often seen as performative measures designed to preserve reputation. This gap between institutional promise and perceived reality contributed to a pervasive sense of mistrust. For many participants, formal feedback mechanisms, equality charters or black, Asian and minority ethnic (BAME) forums did not feel like safe or credible spaces to raise concerns. These findings echo critiques in the wider literature that institutional inclusion efforts may serve to contain rather than challenge inequality. Such initiatives can become tools of organisational control, absorbing dissent while maintaining the appearance of progress.^24 33^ For South Asian staff already managing the risks of racialised and gendered scrutiny, this symbolic inclusion reinforced silence. The gap between values and reality bred mistrust, with some fearing that raising concerns would lead to retaliation instead of reform.

While most participants disengaged from formal structures, a small number turned to trade unions or legal advice, although usually only after facing serious workplace incidents such as bullying or discrimination. These individuals described how access to legal protections shifted their ability to respond to mistreatment. However, such engagement was rare and largely reactive. As the International Labour Organisation (2019) notes, those unaware of their rights are more likely to endure poor conditions in silence.^42^ Similar findings in healthcare have highlighted how legal and organisational protections often fail to translate into actual support, leaving many workers unaware of or reluctant to use formal mechanisms.^43^ It is impossible for the potential of these structures to be realised without better awareness and communication about rights, protections and processes. One participant remarked that she had not known what support was available until she had been forced to seek it: ‘how many rules you don’t know’.

In the NHS context, policies and networks intended to support minoritised staff functioned as containment strategies—absorbing dissent without addressing structural inequality. For staff working in high-pressure clinical environments, this disconnect intensified feelings of isolation and fear of professional repercussions. Rather than validating inclusion, institutional handling of voice became part of the problem.

Nonetheless, for those who did engage with unions or advocacy groups, the experience often proved empowering. Participants described how collective voice made it more difficult for their concerns to be ignored.^25^ Greater awareness of workplace rights not only strengthened individual confidence, but it fostered a sense of accountability across teams. Participants who felt supported were more likely to advocate for colleagues, especially junior staff and nurses, creating informal networks of solidarity and mentorship. Yet this proactive engagement remained exceptional. Many still opted to remain silent or transfer departments rather than trust internal reports systems or union processes. This raises important questions about institutional trust, access and cultural fit. Why do union services remain underused by those most likely to benefit? Why do BAME forums appear ineffective in practice? The answers, our data suggest, lie in the interplay between cultural norms and organisational culture. Fears of being labelled difficult, concerns about professional reputation and low expectations of institutional response discouraged many from seeking formal support. These insights suggest that barriers to access are not simply about availability; psychological safety, credibility and the social cost of voice are equally, if not more, relevant.

To move beyond symbolic inclusion, NHS institutions must invest in building early, visible trust with minoritised staff. Induction processes should go beyond procedural formalities to provide clear guidance on rights, reinforce protections and normalise help-seeking behaviour. Without these proactive measures, even robust support frameworks risk being seen as inaccessible or performative—reinforcing the very culture of silence they are meant to address. In addition, workplaces should invest in sustained mentorship and peer support structures for women of colour; such measures may help reduce isolation, share institutional knowledge and support confidence to exercise voice. NHS organisations should also adopt systematic approaches to recognising and valuing the contributions of minoritised women, including responsive engagement with concerns and visible recognition of professional labour and achievement.

### Strengths and limitations

This study is among the first to explore the intersection of voice, silencing and systemic discrimination for South Asian women healthcare professionals in the NHS during the COVID-19 pandemic. A key strength lies in its focus on a group that has been under-represented in existing organisational and healthcare research, despite evidence that they disproportionately experience inequality. By centring the voices of South Asian women doctors and nurses, the study provides rich insight into how cultural norms, institutional dynamics and workplace hierarchies intersect to shape their experiences of speaking out—or remaining silent—in the face of discrimination, exclusion and professional scrutiny.

Another strength of this research is its in-depth qualitative design, which allowed for the collection of nuanced, lived experiences through semi-structured interviews. Conducted during the pandemic, these interviews captured the heightened pressures faced by NHS staff in real time. This contextual grounding enabled the study to explore how crisis conditions can intensify existing inequalities and silence.

Nevertheless, the study has several limitations. The sample was limited to 27 South Asian women doctors and nurses working within NHS settings. This allowed in-depth qualitative analysis but limits broader generalisability across other clinical roles, specialties and NHS organisations. While our focus on women was intentional to examine intersectional disadvantage, the study does not include the perspectives of male colleagues, managers or senior leaders whose actions and decisions shape workplace cultures, responses to voice and experiences of silencing within the NHS. In addition, although participants were drawn from Pakistani, Bangladeshi and Indian backgrounds, the South Asian healthcare workforce is highly diverse, and experiences may vary across other ethnic, religious and migration groups not represented here. As a cross-sectional study, the findings reflect experiences at a single point in time and cannot capture how voice, confidence or willingness to speak up may change across different stages of NHS careers. Future research could benefit from a multiperspective or longitudinal approach to explore how perceptions of voice, confidence and organisational trust evolve over time.

Recruitment was conducted through snowball sampling via professional gatekeepers, which may have led to a bias towards individuals who were already somewhat engaged or willing to speak about workplace discrimination. Those with more severe or traumatic experiences, or who fear further repercussions, may have opted not to participate.

## Conclusion

This study provides in-depth and original empirical insight into how South Asian women healthcare professionals in the NHS experience voice, silencing and systemic inequality, and how these dynamics became more visible and consequential during the COVID-19 pandemic. Its findings have relevance beyond this specific context, offering broader implications for understanding institutional discrimination, workplace voice and the structural and cultural barriers faced by ethnic minority staff across the healthcare system. Crucially, the study exposes longstanding and often unspoken experiences of exclusion and discrimination that are routinely normalised within everyday organisational practice. In doing so, the study demonstrates that workplace voice is not simply a matter of individual confidence or procedural access, but a rational and institutional phenomenon shaped by power, risk and historical patterns of exclusion. By foregrounding the lived experiences of South Asian women staff, the study highlights the multiple and overlapping mechanisms that suppress voice, including fear of retaliation, hierarchical power dynamics, microaggressions and culturally embedded norms of deference. These findings illustrate how discrimination in healthcare settings is not only structural but also shaped by social expectations and internalised professional risk. The data point to how some participants were able to navigate and resist these challenges through strategies such as legal awareness, union engagement and cultural adaptation, while for others, silence functioned as a necessary strategy for managing risk and protecting themselves within the workplace.

The study’s exploration of the intersection of race, gender, culture and voice among South Asian NHS professionals during a public health crisis contributes new and urgently needed evidence to the growing body of work on inequality and silencing within public sector institutions, particularly in relation to ethnic minority women in healthcare. The findings underscore the importance of intersectional approaches in policy development and institutional reform—approaches that address not only overt forms of discrimination, but also the cumulative effects of exclusion, undervaluing and fear. The study also highlights the importance of transparency in job planning, concern processes and workplace guidance, and of ensuring these systems are accessible and meaningful to all staff.

Further work is needed to understand how these dynamics play out across other ethnic minority groups, professional roles and NHS settings. Longitudinal studies could examine how voice, advocacy and confidence evolve over time, especially as the NHS moves to strengthen equity and inclusion in the period following the pandemic and in preparation for future crises. It is evident from this study that systemic action is required if we are to create an NHS where all professionals, regardless of their background, can speak up without fear, be heard with respect and thrive in their roles. We urge NHS managers to create programmes of leadership training that address power and bias in clinical settings, induction practices that clearly outline the rights, protections and expectations of all staff, and stronger communication around the available support structures to ensure that rights awareness is embedded from the start of employment rather than discovered only in moments of crisis.

## Supplementary material

10.1136/bmjopen-2025-110607 online supplemental file 1

10.1136/bmjopen-2025-110607 online supplemental file 2

## Data Availability

Data are available upon reasonable request.
