# Supplementary material for: Navigating silence and voice: South Asian women healthcare professionals in the UK NHS during COVID-19 and beyond – a qualitative study
Source: BMJ Open. 2026 Mar 12;16(3):e110607. doi: 10.1136/bmjopen-2025-110607 (PMC12983961; doi:10.1136/bmjopen-2025-110607)
Supplement: online supplemental file 1 [file bmjopen-16-3-s001.docx]

**Consolidated criteria for reporting qualitative studies (COREQ): 32-item checklist**

| **No. Item** | **Guide questions/description** | **Reported on Page #** |
| --- | --- | --- |
| **Domain 1: Research team and reﬂexivity** |  |  |
| *Personal Characteristics* |  |  |
| 1. Inter viewer/facilitator | Which author/s conducted the interview or focus group? | First 2 authors; SK and AP; page 11 |
| 2. Credentials | What were the researcher’s credentials? E.g. PhD, MD | First 2 authors PhD; 3^rd^ author MBBS, MD, MSc. (not reported in manuscript) |
| 3. Occupation | What was their occupation at the time of the study? | 1st author Associate Professor; 2^nd^ author Research fellow and 3^rd^ author Consultant/MD (not reported in manuscript) |
| 4. Gender | Was the researcher male or female? | All female authors (not reported in manuscript) |
| 5. Experience and training | What experience or training did the researcher have? | Interviewers were trained in qualitative research methods, had prior experience interviewing healthcare staff and minoritised groups, and completed Good Clinical Practice training. page 10 |
| *Relationship with participants* |  |  |
| 6. Relationship established | Was a relationship established prior to study commencement? | No prior relationship was established between researchers and participants before study commencement. Participants responded to a study invitation circulated via NHS trust channels/staff networks and first contacted the researcher by email; no prior relationship was established. Page 7/8 |
| 7. Participant knowledge of the interviewer | What did the participants know about the researcher? e.g. personal goals, reasons for doing the research | Participants were informed of the study aims, the researchers’ roles, and the purpose of the research through the participant information sheet and consent process. Page 8 |
| 8. Interviewer characteristics | What characteristics were reported about the inter viewer/facilitator? e.g. Bias, assumptions, reasons and interests in the research topic | Yes. Researcher positionality and steps to manage potential bias are described in Methods/Data Analysis (insider/outsider positioning; reflexive memos; peer debrief; steps to mitigate influence on interpretation). page 10 |

| **Domain 2: study design** |  |  |
| --- | --- | --- |
| *Theoretical framework* |  |  |
| 9. Methodological orientation and Theory | What methodological orientation was stated to underpin the study? e.g. grounded theory, discourse analysis, ethnography, phenomenology, content analysis | An inductive qualitative approach using thematic analysis. page 5 |
| *Participant selection* |  |  |
| 10. Sampling | How were participants selected? e.g. purposive, convenience, consecutive, snowball | Participants were recruited using purposive and snowball sampling. Pages 1,2,7 &11 |
| 11. Method of approach | How were participants approached? e.g. face-to-face, telephone, mail, email | Email. Page 8 |
| 12. Sample size | How many participants were in the study? | 27 |
| 13. Non-participation | How many people refused to participate or dropped out? Reasons? | 3 individuals declined and withdrew prior to interview; cited time constraints. Pages 5,11 & 35 |
| *Setting* |  |  |
| 14. Setting of data collection | Where was the data collected? e.g. home, clinic, workplace | Data were collected through interviews online. Page 8 |
| 15. Presence of non-participants | Was anyone else present besides the participants and researchers? | No (not reported in manuscript) |
| 16. Description of sample | What are the important characteristics of the sample? e.g. demographic data, date | Participants were 27 South Asian women (Pakistani, Bangladeshi, Indian) working as doctors and nurses in NHS clinical roles across London, Greater Manchester and Liverpool; recruitment was 2021–2022. Sample characteristics (age/seniority/years of experience etc.) are presented in Table 2 and described in the Results (Demographic data). Page 11 |
| *Data collection* |  |  |
| 17. Interview guide | Were questions, prompts, guides provided by the authors? Was it pilot tested? | A semi-structured interview guide was used/developed (see Methods—Data collection; Appendix 1). Pilot testing: not reported.”- reported on page 9 |
| 18. Repeat interviews | Were repeat inter views carried out? If yes, how many? | No |
| 19. Audio/visual recording | Did the research use audio or visual recording to collect the data? | Audio recorded. Page 8 |
| 20. Field notes | Were ﬁeld notes made during and/or after the interview or focus group? | Yes (not reported in manuscript) |
| 21. Duration | What was the duration of the inter views or focus group? | Interviews lasted approximately 90–110 minutes. page 11 |
| 22. Data saturation | Was data saturation discussed? | Yes — data saturation was discussed. Page 7 |
| 23. Transcripts returned | Were transcripts returned to participants for comment and/or correction? | Not reported |
| **Domain 3: analysis and ﬁndings** |  |  |
| *Data analysis* |  |  |
| 24. Number of data coders | How many data coders coded the data? | 2 – first 2 authors SK and AP **independently… manually coded an initial subset. Page 9** |
| 25. Description of the coding tree | Did authors provide a description of the coding tree? | Yes — a description of the coding structure is provided, supported by a conceptual map (Figure 1). Page 12 |
| 26. Derivation of themes | Were themes identiﬁed in advance or derived from the data? | Themes were inductively derived from the data through thematic analysis. Pages 5,6, &9 |
| 27. Software | What software, if applicable, was used to manage the data? | Not reported |
| 28. Participant checking | Did participants provide feedback on the ﬁndings? | Yes — participants were provided with a summary of the findings and gave positive feedback on the relevance and importance of the study (not reported in manuscript) |
| *Reporting* |  |  |
| 29. Quotations presented | Were participant quotations presented to illustrate the themes/ﬁndings? Was each quotation identiﬁed? e.g. participant number | Yes — anonymised participant quotations were used to illustrate the themes and were labelled using non-identifying descriptors. See findings pages 13-23. |
| 30. Data and ﬁndings consistent | Was there consistency between the data presented and the ﬁndings? | Yes — the findings are grounded in and consistent with the data presented. See pages 11- 23 |
| 31. Clarity of major themes | Were major themes clearly presented in the ﬁndings? | Yes — major themes were clearly presented in the findings. Pages 11-23 |
| 32. Clarity of minor themes | Is there a description of diverse cases or discussion of minor themes? | Yes — variation across participants was discussed, including less common or divergent experiences Pages 13-23 |

The SRQR reporting checklist

For checking that qualitative health research articles can be understood and used by everyone

| 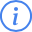 Note |
| --- |
| If you have not used a reporting guideline before, read about [how and why to use them](https:/resources.equator-network.org/about/reporting-guidelines.html) and check whether SRQR is the [most applicable reporting guideline](https:/resources.equator-network.org/reporting-guidelines/srqr/index.html?#applicability) for your work.  Reporting guidelines are most useful when used early in research. When writing a manuscript or application, consider using the [Full Guidance](https:/resources.equator-network.org/reporting-guidelines/srqr/index.html) where you’ll see explanations and examples for each item.  After writing, demonstrate adherence by completing this checklist:   1. Specify where each item is described (see [Note 1](#sec-specify)). 2. Cite this checklist (See [Note 2](#sec-cite)). 3. Include your completed checklist as a supplement when submitting to a journal so that future readers can use it to find information. |

|  | Item Description | Location (or reason for not reporting) |
| --- | --- | --- |
| **Title & Abstract** |  |  |
| [Title](https:/resources.equator-network.org/reporting-guidelines/srqr/items/title.html) | Describe the nature and topic of the study. Identify the study as qualitative or indicate the approach or data collection methods. | Page 1, Line 1 |
| [Abstract](https:/resources.equator-network.org/reporting-guidelines/srqr/items/abstract.html) | Summarise the key elements of the study using the abstract format of the intended publication. | Page 1, line 3 → Page 2, line 16 |
| **Introduction** |  |  |
| [Problem Formulation](https:/resources.equator-network.org/reporting-guidelines/srqr/items/problem-formulation.html) | Describe the problem/phenomenon studied, its significance, relevant theory and empirical work, and gaps in current knowledge. | Page 3, line 5 → Page 6, line 16 |
| [Purpose or research question](https:/resources.equator-network.org/reporting-guidelines/srqr/items/purpose.html) | Describe the purpose of the study and specific objectives or questions. | Page 5, line 22 → Page 6, line 16 |
| **Methods** |  |  |
| [Qualitative approach and research paradigm](https:/resources.equator-network.org/reporting-guidelines/srqr/items/qualitative-approach.html) | Describe your qualitative approach, your guiding theory (if appropriate), and research paradigm, and reasons for your choices. | Page 1, line 8; Page 6, line 18 →Page 7 line 1 |
| [Researcher characteristics and reflexivity](https:/resources.equator-network.org/reporting-guidelines/srqr/items/researcher-characteristics-and-reflexivity.html) | Describe how researchers’ characteristics may influence the research, including personal attributes, qualifications/experience, relationship with participants, assumptions, and/or presuppositions; potential or actual interaction between researchers’ characteristics and the research questions, approach, methods, results and/or transferability. | Page 9, line 23 → Page 10, line 20 |
| [Context](https:/resources.equator-network.org/reporting-guidelines/srqr/items/context.html) | Describe the setting/site(s) in which the study was conducted, why it was selected, and any other salient contextual factors that may influence the study. | Page 3, line 12; Page 7, line 3 |
| [Sampling strategy](https:/resources.equator-network.org/reporting-guidelines/srqr/items/sampling-strategy.html) | Describe how and why research participants, documents, or events were selected; criteria for deciding when no further sampling was necessary, and the rationale for those criteria. | Page 1, line 9; Page 7, line 5 |
| [Ethical issues pertaining to human subjects](https:/resources.equator-network.org/reporting-guidelines/srqr/items/ethics.html) | Describe any approval by an appropriate ethics review board and participant consent, or explain any lack thereof. Describe any other confidentiality and data security issues. | Page 7, line 16 - 22 |
| [Data collection methods](https:/resources.equator-network.org/reporting-guidelines/srqr/items/data-collection-methods.html) | Describe the types of data collected; details of data collection procedures including (as appropriate) start and stop dates of data collection and analysis, iterative process, triangulation of sources/methods, and modification of procedures in response to evolving study findings. Describe your rationale for these choices. | Page 8, line 2 → Page 9, line 13; Table1 |
| [Data collection instruments and technologies](https:/resources.equator-network.org/reporting-guidelines/srqr/items/data-collection-instruments.html) | Describe any instruments (e.g., interview guides, questionnaires) and devices (e.g., audio recorders) used for data collection; describe if/how the instrument(s) changed over the course of the study. | Page 8, line 22 → Page 9, line 13; Table 1 and Appendix 1. |
| [Units of study](https:/resources.equator-network.org/reporting-guidelines/srqr/items/units-of-study.html) | Describe the number and relevant characteristics of participants, documents, or events included in the study. Describe the level of participation. | Page 11, line 12 - 20; Table 2 |
| [Data processing](https:/resources.equator-network.org/reporting-guidelines/srqr/items/data-processing.html) | Describe the methods for processing data prior to and during analysis, including transcription, data entry, data management and security, verification of data integrity, data coding, and anonymisation / deidentification of excerpts. | Page 7, line 9; Page 9, line 16 |
| [Data analysis](https:/resources.equator-network.org/reporting-guidelines/srqr/items/data-analysis.html) | Describe the process by which inferences, themes, etc. were identified and developed, including the researchers involved in data analysis; usually references a specific paradigm or approach. Describe why you chose this process. | Page 9, line 15 → Page 10 line 2 |
| [Techniques to enhance trustworthiness](https:/resources.equator-network.org/reporting-guidelines/srqr/items/trustworthiness.html) | Describe any techniques to enhance trustworthiness and credibility of data analysis,(e.g., member checking, triangulation, audit trail). Describe why you chose these techniques. | Page 9, line 15 → Page 10 line 2 |
| **Results** |  |  |
| [Synthesis and interpretation](https:/resources.equator-network.org/reporting-guidelines/srqr/items/synthesis-and-interpretation.html) | Describe the main findings (e.g., interpretations, inferences, and themes); might include development of a theory or model, or integration with prior research or theory. | Page 12, line 2 → Page 23, line 16; Figure 1 |
| [Links to empirical data](https:/resources.equator-network.org/reporting-guidelines/srqr/items/links-to-empirical-data.html) | Provide evidence (e.g., quotes, field notes, text excerpts, photographs) to substantiate analytic findings. | Page 12 line 17 → Page 23, line 16 |
| **Discussion** |  |  |
| [Integration with prior work, implications, transferability, and contribution(s) to the field](https:/resources.equator-network.org/reporting-guidelines/srqr/items/integration-with-prior-work.html) | Summarize the main findings, explain how findings and conclusions connect to, support, elaborate on, or challenge conclusions of earlier scholarship; discuss the scope of application/generalizability; identify unique contribution(s) to scholarship in a discipline or field. | Page 23, line 18 → Page 34, line 22 |
| [Limitations](https:/resources.equator-network.org/reporting-guidelines/srqr/items/limitations.html) | Discuss the trustworthiness and limitations of findings | Page 2 line 8 → Page 3, line 2; Page 41 line 15 |
| **Other** |  |  |
| [Conflicts of interest](https:/resources.equator-network.org/reporting-guidelines/srqr/items/conflicts-of-interest.html) | Describe any potential sources of influence or perceived influence on study conduct and conclusions. Describe how these were managed. | Page 38, line 16 |
| [Funding](https:/resources.equator-network.org/reporting-guidelines/srqr/items/funding.html) | Describe sources of funding and other support. Describe the role of funders in data collection, interpretation, and reporting. | Page 38, line 10 |

## 1 How to specify where content is

Tell the reader where they can find information. E.g.,

- Results; paragraph 2
- Methods, Participants; paragraphs 1 & 2.
- Table 3
- Supplement B, para. 4

If you have chosen not to describe an item, explain why. You can do this in the checklist, or as a note below it.

You can describe items in the article body, or in tables, figures, or supplementary materials, and should prioritize items you feel are most important to your intended audience. The order of items in your manuscript does not need to match the order of items in this checklist. You can decide how best to structure your work.

## 2 How to cite

Describe how you used SRQR at the end of your Methods section, referencing the resources you used e.g.,

‘We used the SRQR reporting guideline(1) to draft this manuscript, and the SRQR reporting checklist(2) when editing, included in supplement A’

If you use a reporting checklist, remember to include it as a supplement when publishing so that readers can easily find information and see how you have interpreted the guidance.

1. O’Brien BC, Harris IB, Beckman TJ, Reed DA, Cook DA. Standards for reporting qualitative research: A synthesis of recommendations. Academic Medicine [Internet]. 2014 Sep;89(9):1245–51. Available from: <https://journals.lww.com/academicmedicine/fulltext/2014/09000/Standards_for_Reporting_Qualitative_Research__A.21.aspx>

2. O’Brien BC, Harris IB, Beckman TJ, Reed DA, Cook DA. The SRQR reporting checklist. In: Harwood J, Albury C, Beyer J de, Schlüssel M, Collins G, editors. The EQUATOR network reporting guideline platform [Internet]. The UK EQUATOR Centre; 2025. Available from: [https:/resources.equator-network.org/reporting-guidelines/srqr/srqr-checklist.docx](https://https:/resources.equator-network.org/reporting-guidelines/srqr/srqr-checklist.docx)
