# Supplementary material for: Navigating silence and voice: South Asian women healthcare professionals in the UK NHS during COVID-19 and beyond – a qualitative study
Source: BMJ Open. 2026 Mar 12;16(3):e110607. doi: 10.1136/bmjopen-2025-110607 (PMC12983961; doi:10.1136/bmjopen-2025-110607)
Supplement: online supplemental file 2 [file bmjopen-16-3-s002.docx]

**Interview Questions**

The study used a semi-structured interview guide organised around key themes. Questions were used flexibly to follow participants’ accounts, and not all questions were asked in every interview.

**Establishing Rapport**

Before we begin, it would be nice if you could tell me a little bit about yourself.

Age:

Occupation (grade):

Place of work:

Ethnic background:

Birthplace:

How long have you been in the UK (if not born in the UK)?

Marital status (if applicable):

The number of children (if applicable):

**(1) Understanding the Participant**

**(a) Household and Family Life**

- Can you tell us a bit about your household and family life?
- How many people are in your household?
- How do you feel about this arrangement?
- Has anything changed since the pandemic started?
- Do you have children? If so, how did you manage childcare/homeschooling during the COVID -19 lockdowns?
- If married: How did you divide the house chores before the pandemic? Has it changed?
- Do you have family support?
- How do you see yourself within your family context? What do you think your role and responsibilities are? Has that changed with COVID -19?
- What would have supported you better during these times?
- Has COVID- 19 impacted your household and family life? How so?

**(b) Daily life**

- Has your daily routine changed due to COVID? Please tell me about what’s different compared with before.
- What sorts of activities did you do pre-COVID? [e.g., work, school, leisure] Can you explain how your weekly routine looked like before?
- Who did you mostly spend time with? And now?

**(c) Social-economic Profile**

- Where did you grow up?
- Can you tell me a bit about your parents? (Prompts: Are your parents in the UK? What are your parents’ occupations?)
- Growing up, what sorts of things did you enjoy doing?
- How would you describe yourself; do you identify with a class background?
- Who do you support financially?
- COVID -19 has impacted people in different ways. Some people were able to save money because of not going out much, for example. Others lost jobs or had to support family members who lost their jobs. Has the Covid-19 pandemic impacted your financial situation?

**(d) Self-identification**

These self-identification questions will vary slightly depending on how comfortable the participant feels talking about their ethnic background. It could be influenced by how long the person has been in the UK, or if they were born here, or any other variable that might come up during the conversation. Here the interviewer should aim to get a sense of how the interviewee sees herself.

- How would you describe yourself? What would you say is your Ethnic group?
- What are you most proud of about your background?

**(e) Participant’s Trajectory**

This section aims to understand the pathway to working and becoming a professional working women in the health sector. By giving us your story in your own words, we will understand which elements of your experience are significant. This again will assist us in understanding social-economic and gendered practices. Following this we will ask questions regarding your work environment and responses to Covid-19.

- Can you tell me how you came to do this job? (Here, we are looking for meaningful stories of difficulties or easiness as well as hardship and resilience.)
- How do you feel about your chosen path?
- From when you started until now, have you fulfilled your careers goals?
- What could help you in your path?
- Has Covid changed anything in the way you think about your work?

**2: Understanding the Workplace**

**(a) Daily Work Routine**

- Can you walk me through your daily work routine?
- Who do you interact with daily?
- How has it changed/ adapted to the Covid pandemic?
- How do you feel about that?
- What would you do differently?

**(b) Interaction with Patients**

- Can you tell us a bit about your interaction with patients?
- Have you ever felt that you were treated differently because of your ethnicity?
- Has anything changed with Covid?

**(c) Interaction with Co-Workers**

- What about interaction with co-workers?
- Have you ever felt that you were treated differently because of ethnicity?
- Would you change anything in your position and interactions within the organization?

**3) Understanding Covid Response**

**(a) Covid in the Workplace**

- Can you tell us your work journey since the beginning of the pandemic?

Can you walk us through how your place reacted or implemented the Covid preventive measures?

- Were you ever assigned to work in a Covid intensive care unit?
- Did you have to wear full PPE?
- What is your opinion on the Covid response at your workplace?
- What would you have done differently?

**(b) Covid in your Personal/Social space**

- Has anyone in your household had Covid?
- What was the impact on you?
- Did you have any support during the pandemic? (Do you have a support network)
- Are you part of a larger community? (For example, ethnic or religious community)
- If you are, do you agree with the way they behaved during more extreme lockdown restrictions?
- What do you think of vaccines?
- As a woman, is there anything that could have helped you?

Thank you very much for your time and the information you shared today.
