# Supplementary material for: Navigating silence and voice: South Asian women healthcare professionals in the UK NHS during COVID-19 and beyond – a qualitative study
Source: BMJ Open. 2026 Mar 12;16(3):e110607. doi: 10.1136/bmjopen-2025-110607 (PMC12983961; doi:10.1136/bmjopen-2025-110607)
Supplement: Supplementary file 3 [file bmjopen-2025-110607.reviewer_comments.pdf]

PEER REVIEW HISTORY

BMJ Open publishes all reviews undertaken for accepted manuscripts. Reviewers are asked to complete a checklist review form and are provided with free text boxes to elaborate on their assessment. These free text comments are reproduced below.

ARTICLE DETAILS

Title (Provisional)

Navigating Silence and Voice: South Asian Women Healthcare Professionals in the UK NHS During COVID-19 and Beyond: A Qualitative Study.

Authors

Kauser, Saleema; defigueiredosilva, Ana Paula; Pandya, Bhavna K

VERSION 1 - REVIEW

**Reviewer**	**1**
**Name**	**Nowrouzi-Kia, Behdin**
**Affiliation**	**University of Toronto, Occupational Science and Occupational Therapy**
**Date**	**06-Oct-2025**
**COI**	**None**

Thank you for the opportunity to review this paper. The authors have conducted a qualitative study to examine the experience of south Asian female healthcare professionals in the United Kingdom. This is an interesting study that reviews the experiences of healthcare workers in the United Kingdom through an intersectional approach. I believe the paper has the potential to contribute to our understanding how to improve the work environment of health care workers. However, there are significant issues that must be addressed before the paper is accepted. I have structured my feedback for each section of the paper. I welcome the opportunity to review the revised paper.

Abstract

It would be helpful to include the location (country) where the study is conducted as not all readers will know what the NHS. The journal has a global reach and it would be beneficial to also include it the title as well.

Introduction

The section is well structured but lacks signification content that I believe will strengthen the quality of the paper. The author should further expand on the knowledge gaps that the current study will address. It would also be helpful to provide some theoretical discussion of

how the proposed work will align with. This is particularly important given that the authors have selected a qualitative approach using an interpretivist and inductive approach.

Methods

The authors should include the COREQ checklist and address all the sections of the checklist. This is going to be helpful in situating the interview with the participants of the study as well as other aspects of the study. I would like some additional details about the data analysis for the study.

Results

This section would benefit from the inclusion of a concept map to show the link between the primary thematic domains and codes. It would also be beneficial to show the relationship between the data in a visual manner.

Discussion

This section is well written and the authors have done a sufficient job linking the findings to the extant literature. I would invite them to delver further and provide recommendations for workers and workplaces for implementing the findings into practice. I'd also like to see an expansion of the study limitations and the conclusion.

**Reviewer**	**2**
**Name**	**Karavadra, Babu**
**Affiliation**	**Norfolk and Norwich University Hospital, University of Liverpool**
**Date**	**14-Nov-2025**
**COI**	**None**

Overall: the authors have attempted to discuss the experiences of south Asian women healthcare professionals with regards to 'silence and voice' during the covid-19 pandemic and beyond. There are some very interesting findings. However, having read the manuscript twice, I am unclear on how their findings relate to the covid-19 pandemic and how this adds to the literature base from this perspective. In my opinion, this study could have been done at any other time, apart from the covid-19 pandemic and the findings may still have been the same. I am struggling to see how the pandemic relates to the contextualisation of the findings. I am unsure why the authors have included other literature within their own findings section, and then also include a discussion section with other literature. Generally, the results section should only include findings from the authors study, and any comparison with the existing literature should be discussed in the discussion section.

Is the research question or study objective clearly defined?

In the abstract, whilst the research question is defined, it is not clear from the abstract how this relates specifically to the covid-19 pandemic

Is the abstract balanced and complete?

The findings section of the abstract is clear and concise. However, based on the abstract, I am still not clear on how the study relates to the covid-19 pandemic and why the authors decided to do this during that time. The abstract conclusion does not mention the covid-19 pandemic either.

Background section

The section contains a lot of useful and important literature. However, the background section is quite long and unfocussed at times. There is no mention of the research question at the end of the background section- this would be useful as it will help tie the existing literature with any gaps.

Is the study design appropriate to answer the research question?

The study design is appropriate for the research question and is detailed clearly. Good description of the study methods. The creation of the interview topic guide does not mention any PPIE involvement. There is good discussion about reflexivity- an important consideration for any qualitative research.

Are the methods described sufficiently to allow the study to be repeated?

Yes.

Are research ethics (e.g. participant consent, ethics approval) addressed appropriately?

Ethical approval is mentioned, but the ethics board is not mentioned. There is a lot of detail in the methods section, however, there is no mention of specific ethical considerations that the authors may have thought about- for example, if participants found talking about their experiences, then how would the researchers handle this?

Are the outcomes clearly defined?

No. I am not clear how this study relates to the covid-19 pandemic and therefore the findings are difficult to contextualise.

If statistics are used are they appropriate and described fully?

Not applicable

Are the references up-to-date and appropriate?

Yes. There is a through review of the existing literature base.

Do the results address the research question or objective?

The results section provides some useful insights. However, I am concerned about the length of this article and there are several areas within the results section that merges the existing

literature base with the findings. It would have been clearer to only include the results in the results section and then have a discussion section that amalgamates the existing literature base for comparison. Currently, the results section is considerably long, and the reader may easily forget the pertinent points from the results section.

Page 10 Lines 22-24: in the results section, the authors have discussed existing literature. This belongs in the discussion section.

Page 14 lines 15-17: I am not sure what the authors mean by 'dramatically more patients' This is not evidenced with a participant quote.

Is the supplementary reporting complete (e.g. trial registration; funding details; CONSORT, STROBE or PRISMA checklist)?

Not applicable

To the best of your knowledge is the paper free from concerns over publication ethics (e.g. plagiarism, redundant publication, undeclared conflicts of interest)?

Yes.

Is the standard of written English acceptable for publication?

Yes

VERSION 1 - AUTHOR RESPONSE

Reviewer: 1: **Prof. Behdin Nowrouzi-Kia, University of Toronto**

Comments to the Author:
Thank you for the opportunity to review this paper. The authors have conducted a qualitative study to examine the experience of south Asian female healthcare professionals in the United Kingdom. This is an interesting study that reviews the experiences of healthcare workers in the United Kingdom through an intersectional approach. I believe the paper has the potential to contribute to our understanding how to improve the work environment of health care workers. However, there are significant issues that must be addressed before the paper is accepted. I have structured my feedback for each section of the paper. I welcome the opportunity to review the revised paper.

We thank the reviewer for their careful reading of the manuscript and for their constructive and thoughtful comments. We appreciate their recognition of the study's potential contribution and their detailed feedback, which has helped us to strengthen and clarify the manuscript. We have addressed each comment in detail below.

Abstract:
It would be helpful to include the location (country) where the study is conducted as not all readers will know what the NHS. The journal has a global reach and it would be beneficial to also include it the title as well.
Response:

We have revised both the title and the abstract to explicitly state that the study was conducted in the United Kingdom, so that the NHS context is clear to readers unfamiliar with the UK healthcare system.

Introduction:
The section is well structured but lacks signification content that I believe will strengthen the quality of the paper. The author should further expand on the knowledge gaps that the current study will address.

Response:
We have revised the Introduction to more clearly identify and expand on the knowledge gaps addressed by this study. We now emphasise the limited empirical research focusing on South Asian women healthcare professionals lived experiences of voice, silence, and risk within the NHS, and the lack of work examining how these dynamics are experienced and navigated during periods of heightened organisational pressure such as the COVID-19 pandemic.

It would also be helpful to provide some theoretical discussion of how the proposed work will align with. This is particularly important given that the authors have selected a qualitative approach using an interpretivist and inductive approach.

Response:
We have strengthened the theoretical discussion in the Introduction by explicitly situating the study within interpretivist and inductive qualitative traditions. We now explain how this approach allows for an in-depth exploration of meaning-making, institutional context, and power relations shaping voice and silence, and why it is appropriate for examining South Asian women's experiences within the NHS during the COVID-19 pandemic.

Methods
The authors should include the COREQ checklist and address all the sections of the checklist. This is going to be helpful in situating the interview with the participants of the study as well as other aspects of the study.

Response: ***We have included the COREQ checklist as Supplemental Material and ensured that all relevant sections of the checklist are addressed within the manuscript. This includes clearer reporting of researcher characteristics, participant recruitment, data collection procedures, and reflexivity, in line with COREQ guidance***

I would like some additional details about the data analysis for the study.

Response:
We have expanded the Data Analysis section to provide greater detail on the analytical process. Specifically, we now describe the stages of inductive thematic analysis, including transcript familiarisation, line-by-line coding, development and refinement of a coding template, inter-coder discussion and agreement, use of reflexive memos, and peer debriefing, as well as how consensus on themes and subthemes was reached.

Results
This section would benefit from the inclusion of a concept map to show the link between the

primary thematic domains and codes. It would also be beneficial to show the relationship between the data in a visual manner.

Response:
We have added a visual concept map illustrating the relationships between the primary thematic domains identified in the analysis. This has been included as Figure 1 in the Results section and is referenced in the opening paragraph describing the four main themes

Discussion
This section is well written and the authors have done a sufficient job linking the findings to the extant literature. I would invite them to delver further and provide recommendations for workers and workplaces for implementing the findings into practice. I'd also like to see an expansion of the study limitations and the conclusion.

Response:
We thank the reviewer for this constructive feedback. In response, we have strengthened the practical implications of the study by more explicitly articulating recommendations for workplaces and organisations within the Discussion and Conclusion sections. These include the need for sustained mentorship and peer support structures for women of colour, clearer communication around rights and protections, transparent job planning and complaints processes, and more responsive organisational engagement with concerns raised by minoritised staff. These recommendations are directly grounded in participants' accounts and the themes identified in the analysis.

We have also expanded the Strengths and limitations section to provide a clearer and more detailed discussion of the study's scope and constraints. This includes reflection on the qualitative design, sample composition, focus on South Asian women, use of snowball sampling, and the absence of perspectives from managers and organisational leaders. We believe these revisions clarify both the contribution and the boundaries of the study while remaining appropriate to the length and format of the manuscript.

We have revised the Conclusion to strengthen its articulation of the study's overall contribution and implications, while remaining consistent with the scope, focus, and length of the manuscript. The revised Conclusion now more clearly situates the findings within the context of the COVID-19 pandemic as a period in which pre-existing constraints on voice became more visible and consequential, and it clarifies the relevance of the study for understanding voice, silence, and institutional inequality within the NHS and beyond .Given the length and structure of the paper, we have retained a concise Conclusion that aligns with the journal's conventions and avoids introducing new material beyond what is supported by the data and analysis presented in the manuscript.

<u>Reviewer: 2 Dr. Babu Karavadra, Norfolk and Norwich University Hospital</u>

Comments to the Author:
Overall: the authors have attempted to discuss the experiences of south Asian women healthcare professionals with regards to 'silence and voice' during the covid-19 pandemic and beyond. There are some very interesting findings. However, having read the manuscript

twice, I am unclear on how their findings relate to the covid-19 pandemic and how this adds to the literature base from this perspective. In my opinion, this study could have been done at any other time, apart from the covid-19 pandemic and the findings may still have been the same. I am struggling to see how the pandemic relates to the contextualisation of the findings.

Response:
We thank the reviewer for raising this important point. We have clarified throughout the manuscript how COVID-19 functions not as the origin of the identified dynamics, but as a critical context in which pre-existing constraints on voice became more visible, intensified, and consequential.

We have made explicit in the Background, Results, and Discussion sections, that the pandemic heightened organisational pressure, uncertainty, and risk, narrowing opportunities to raise concerns and amplifying the consequences of speaking up. Participants' accounts demonstrate that COVID-19 altered the stakes of voice by intensifying workload demands, safety risks, redeployment practices, and regulatory scrutiny, particularly for South Asian women who were already navigating racialised and gendered power dynamics.

We have therefore positioned COVID-19 as a revelatory and amplifying context that exposed structural and cultural inequalities that might otherwise remain less visible in routine conditions, rather than as a singular causal factor. This framing is reflected in the Results, where participants explicitly situate their experiences of silence, risk, and adaptation within pandemic-specific conditions, and in the Discussion, where we show how crisis conditions sharpen existing patterns of institutional silencing. We believe this clarification strengthens the contribution of the study to the COVID-19 literature while also demonstrating its relevance beyond the pandemic period.

I am unsure why the authors have included other literature within their own findings section, and then also include a discussion section with other literature. Generally, the results section should only include findings from the authors study, and any comparison with the existing literature should be discussed in the discussion section.

Response:
We thank the reviewer for this helpful clarification. In response, we have revised the manuscript to ensure clearer separation between the Results and Discussion sections. The Results section has been edited so that it now focuses exclusively on findings derived from participants' accounts, illustrated through themes and quotations from the data. Comparisons with, and interpretation in relation to, the existing literature have been moved to and consolidated within the Discussion section. We believe this revision improves the structure and readability of the manuscript and aligns it more closely with conventional reporting standards.

Is the research question or study objective clearly defined?
In the abstract, whilst the research question is defined, it is not clear from the abstract how this relates specifically to the covid-19 pandemic

Response:
Thank you for this comment. We have revised the Abstract to make explicit that the research question is examined within the context of the COVID-19 pandemic, by stating

that participants' experiences were explored during COVID-19 and that the findings reflect how pandemic conditions shaped experiences of voice, silence, and risk.

Is the abstract balanced and complete?
The findings section of the abstract is clear and concise. However, based on the abstract, I am still not clear on how the study relates to the covid-19 pandemic and why the authors decided to do this during that time. The abstract conclusion does not mention the covid-19 pandemic either.

Response:
Thank you for this comment. We would like to clarify that we conducted the study during COVID-19, which functioned not only as a background condition but as a period of heightened institutional strain. While the discrimination and silencing described by participants often pre-dated the pandemic, our analysis suggests that COVID-19 exposed and amplified these dynamics by narrowing opportunities to raise concerns, increasing workloads and personal risk, and heightening fear of retaliation. From an interpretive qualitative perspective, we understand participants' retrospective reflections during the pandemic as analytically meaningful sense-making rather than distortion. We therefore treat COVID-19 as a contextual amplifier of existing organisational dynamics rather than as a standalone analytical theme or causal explanation. We have revised the Abstract to make this clearer and amended the conclusion to explicitly mention the COVID-19 pandemic.

Background section
The section contains a lot of useful and important literature. However, the background section is quite long and unfocussed at times. There is no mention of the research question at the end of the background section- this would be useful as it will help tie the existing literature with any gaps.

Response:
Thank you for this feedback. We have revised the Background section to improve focus and readability by removing repeated material, tightening wording throughout, and streamlining sections that overlapped in content. We deleted text that repeated similar points about staff voice and organisational pressures and reduced broader contextual detail that was not directly required to introduce the study's rationale. We also re-structured the Background so that related issues are grouped more clearly, including separating the discussion of pandemic-related organisational pressures from the discussion of cultural and institutional mechanisms of silencing.

We have also now made the research question explicit within the final part of the Background to directly connect the reviewed literature and the identified gap to the purpose of the study. Specifically, the Background now introduces the research question in the concluding section as a new paragraph: "Against this broader policy and institutional scenario, we asked: how did South Asian women healthcare professionals experience and navigate voice, silence and risk within the NHS during the COVID-19 pandemic?" This is followed by a short linking explanation that situates the question within the study design and pandemic context.

Is the study design appropriate to answer the research question?
The study design is appropriate for the research question and is detailed clearly. Good description of the study methods. The creation of the interview topic guide does not mention any PPIE involvement. There is good discussion about reflexivity- an important consideration for any qualitative research.

Response:
Thank you for highlighting this. We have now added a "Patient and public involvement" subsection in the Methods stating that patients and the public were not involved in the design, conduct, reporting, or dissemination of this (as required by the journal) As the interview topic guide formed part of the study design, it was likewise developed by the research team without patient/public involvement.

Are the methods described sufficiently to allow the study to be repeated?
Yes.

Are research ethics (e.g. participant consent, ethics approval) addressed appropriately?
Ethical approval is mentioned, but the ethics board is not mentioned. There is a lot of detail in the methods section, however, there is no mention of specific ethical considerations that the authors may have thought about- for example, if participants found talking about their experiences, then how would the researchers handle this?

Response:
Thank you for this comment. We have updated the Methods to name the ethics bodies that approved the study. We now state that approval was obtained from the University Research Ethics Committee and the Health Research Authority, and that Trust Research Directorate approval was confirmed for each participating NHS trust before recruitment began.

We have also added a sentence to explain how we managed the risk of distress, given the sensitivity of discussing workplace discrimination and silencing. The Methods now notes that participants could pause the interview, decline to answer any questions, or stop the interview if needed

Are the outcomes clearly defined?
No. I am not clear how this study relates to the covid-19 pandemic and therefore the findings are difficult to contextualise.

Response
The study *outcomes concern institutional voice and silencing, with COVID-19 providing the context in which these dynamics came to light in the participants' narratives, rather than being a separate analytical outcome*

If statistics are used, are they appropriate and described fully?
Not applicable

Are the references up-to-date and appropriate?
Yes. There is a through review of the existing literature base.

Do the results address the research question or objective?

The results section provides some useful insights. However, I am concerned about the length of this article and there are several areas within the results section that merges the existing literature base with the findings. It would have been clearer to only include the results in the results section and then have a discussion section that amalgamates the existing literature base for comparison. Currently, the results section is considerably long, and the reader may easily forget the pertinent points from the results section.

Response: ***Thank you for this feedback. We agree that the Results section should present our study data only, with comparisons to other published work reserved for the Discussion. We have therefore revised the Results to remove references to the existing literature and any interpretive comparison, and we have moved that material into the Discussion where appropriate. We have also tightened the Results by focusing on the key themes and the most relevant supporting quotations, and by keeping descriptive participant information at the start of the Results to help the reader follow the findings. These changes were made to reduce length, avoid mixing evidence with interpretation, and make the main findings easier to retain.***

Page 10 Lines 22-24: in the results section, the authors have discussed existing literature. This belongs in the discussion section.

Response:
We have removed this from the results section

Page 14 lines 15-17: I am not sure what the authors mean by 'dramatically more patients' This is not evidenced with a participant quote.

Response
We have removed the word dramatically

Is the supplementary reporting complete (e.g. trial registration; funding details; CONSORT, STROBE or PRISMA checklist)?
Not applicable

To the best of your knowledge is the paper free from concerns over publication ethics (e.g. plagiarism, redundant publication, undeclared conflicts of interest)?
Yes.

Is the standard of written English acceptable for publication?
Yes

Reviewer: 1
If you have selected 'Yes' above, please provide details of any competing interests.: Not applicable

Reviewer: 2
If you have selected ‘Yes’ above, please provide details of any competing interests.: not applicable

VERSION 2 - REVIEW

**Reviewer**	**1**
**Name**	**Nowrouzi-Kia, Behdin**
**Affiliation**	**University of Toronto, Occupational Science and Occupational Therapy**
**Date**	**15-Feb-2026**
**COI**	

Thank you for submitting your revised paper. You have adequately addressed my feedback, and I am recommending that the paper be accepted for publication.
